# The impact of dietary fibers on *Clostridioides difficile* infection in a mouse model

**DOI:** 10.3389/fcimb.2022.1028267

**Published:** 2022-11-09

**Authors:** Zhengjie Wu, Qiaomai Xu, Qiangqiang Wang, Yunbo Chen, Longxian Lv, Beiwen Zheng, Ren Yan, Huiyong Jiang, Jian Shen, Shuting Wang, Kaicen Wang, Jiafeng Xia, Shengyi Han, Lanjuan Li

**Affiliations:** ^1^ State Key Laboratory for Diagnosis and Treatment of Infectious Diseases, National Clinical Research Center for Infectious Diseases, National Medical Center for Infectious Diseases, Collaborative Innovation Center for Diagnosis and Treatment of Infectious Diseases, The First Affiliated Hospital, Zhejiang University School of Medicine, Hangzhou, China; ^2^ Jinan Microecological Biomedicine Shandong Laboratory, Jinan, China

**Keywords:** *Clostridioides difficile*, dietary fiber, pectin, microbiota, short-chain fatty acids

## Abstract

Diets rich in fiber may provide health benefits and regulate the gut microbiome, which affects the immune system. However, the role of dietary fiber in *Clostridioides difficile* infection (CDI) is controversial. Here, we investigated the use of fermentable fibers, such as inulin or pectin, to replace the insoluble fiber cellulose to explore how dietary fiber affects *C. difficile*-induced colitis in mice through intestinal microecology and metabolomics. Using *C. difficile* VPI 10463, we generated a mouse model of antibiotic-induced CDI. We evaluated disease outcomes and the microbial community among mice fed two fermentable fibers (inulin or pectin) versus the insoluble fiber cellulose. We analyzed and compared the gut microbiota, intestinal epithelium, cytokine levels, immune responses, and metabolites between the groups. Severe histological injury and elevated cytokine levels were observed in colon tissues after infection. Different diets showed different effects, and pectin administration protected intestinal epithelial permeability. Pectin also steadily increased the diversity of the microbiome and decreased the levels of *C. difficile*-induced markers of inflammation in serum and colonic tissues. The pectin group showed a higher abundance of *Lachnospiraceae* and a lower abundance of the conditionally pathogenic *Enterobacteriaceae* than the cellulose group with infection. The concentration of short-chain fatty acids in the cecal contents was also higher in the pectin group than in the cellulose group. Pectin exerted its effects through the aryl hydrocarbon receptor (AhR) pathway, which was confirmed by using the AhR agonist FICZ and the inhibitor CH2223191. Our results show that pectin alters the microbiome and metabolic function and triggers a protective immune response.

## Introduction

*Clostridioides difficile* infection (CDI) causes more than 70% of healthcare-associated gastrointestinal infections, with outcomes ranging from diarrhea, colitis, and severe toxic megacolon (Smits et al., 2016). The stable gut microbial community is a natural barrier against *C. difficile* (Ling et al., 2022). CDI may be caused by disruption of the resident intestinal microbiota. Antibiotic therapy is essential for treating bacterial infections. Most notably, repeated exposure of the intestinal microbiota to antibiotics eliminates commensal bacteria from the intestinal ecology and provides opportunities for pathogens, such as *C. difficile*, to colonize and proliferate. The metabolites of the intestinal flora, including short-chain fatty acids (SCFAs) and bile acids (Kochan et al., 2018), have been associated with *C. difficile*-induced disease. Therefore, diet may influence the incidence and severity of CDI. The gut microbiota and *C. difficile* metabolic interactions determine *C. difficile* fitness (Ng et al., 2013; Buffie et al., 2015; Yan et al., 2021), and reductions in the levels of dietary microbiota-related metabolites cause colon inflammation (Earle et al., 2015).

The Western diet includes high consumption of fatty foods and low consumption of naturally fiber-rich grains, fruits, and vegetables. Low dietary fiber intake may lead to impaired intestinal health and an increased prevalence of chronic inflammatory diseases. Dietary fiber can be broadly classified as insoluble (e.g., cellulose) or soluble (e.g., pectin, inulin), and the soluble fiber is readily fermented by intestinal bacteria to produce SCFAs (Singh et al., 2019). The transformation of dietary fiber into available nutrients is one of the main benefits that the gut microbiota provides to the host. The mechanisms underlying the association of dietary fiber with the development of intestinal inflammation and immune-related diseases have not been fully studied. A diet low in dietary fiber alters the gut microbiota and its metabolism, thereby disrupting host-microbiota interactions (Sonnenburg and Sonnenburg, 2014). Studies have reported conflicting results regarding the relationship between diet and CDI. Some have shown that dietary fiber can alleviate antibiotic-induced CDI (Hryckowian et al., 2018). Other studies suggest that dietary fiber may promote antibiotic-related ecological dysregulation and long-term *C. difficile* carriage (Bhute et al., 2022). Pectin may play a role by regulating the intestinal microbiota composition and T-cell responses (Bernard et al., 2015; Ishisono et al., 2019; Wu et al., 2019). Understanding the effects and potential mechanisms through which dietary carbohydrates influence CDI may offer useful insights into pathogenesis.

Here, we evaluated the effects of soluble dietary fibers (pectin and inulin) on CDI and described the flora diversity and metabolic structure. Thus, we tested whether soluble fiber is more beneficial than unfermentable fiber during CDI due to its ability to act as an SCFA precursor. We report that the dietary soluble fiber pectin ameliorates colitis in a *C. difficile-*related model. The effect of the pectin diet was also explored by targeting the aryl hydrocarbon receptor (AhR) pathway through which it may exert its protective effect.

## Methods

### Model of infection

**Figure 1:**
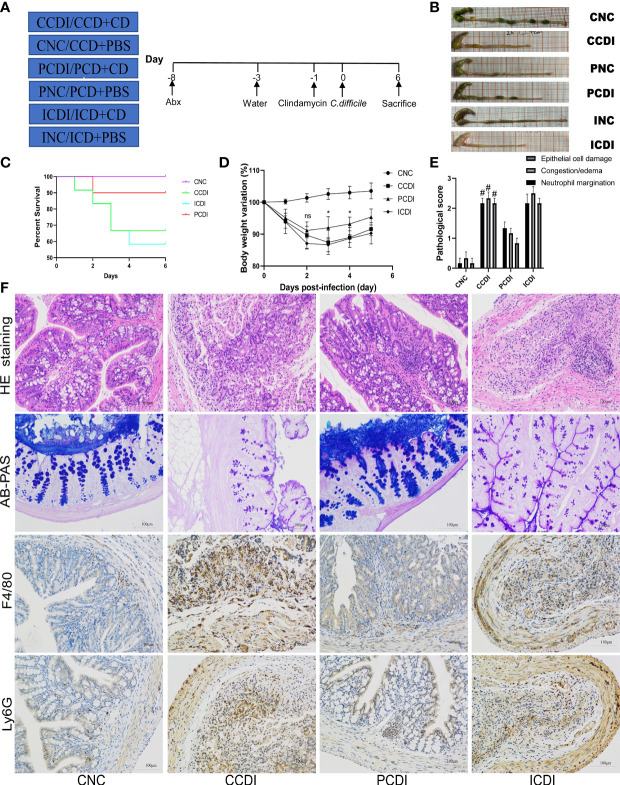
A pectin diet protects mice from *C*. *difficile* infection. **(A)** Experimental design diagram; CDI modeling after two weeks of dietary intervention. **(B)** Representative images of the colon. **(C)** Body weight change curves of the mice. **(D)** Survival curves of different groups. **(E, F)** Pathological analysis and histopathological scoring of colon tissue of the mice by H&E staining, AB-PAS staining and immunohistochemistry (F4/80 and Ly6G). ^#^, CCDI vs PCDI, *P*<0.05; ns, differences are not significant. CNC, cellulose diet with control group; CCDI, cellulose diet with *C*. *difficile* infection; PNC, pectin diet with control group; PCDI, pectin diet with *C*. *difficile* infection; INC, inulin diet with control group; ICDI, inulin diet with *C*. *difficile* infection.

*C. difficile* strain VPI 10463 (ATCC 43255) was cultivated in Difco cooked meat medium (BD Diagnostic Systems, USA) in an anaerobic atmosphere. C57BL/6 male mice (6-8 weeks) were housed and fed a standard laboratory diet for one week (**Figure 1A**). The mice were then randomly divided: two groups were fed a cellulose control diet (CNC=8 and CCDI=12), and the remaining groups were fed a pectin diet (PCDI=12, PNC=8) and an inulin diet (ICDI=12, INC=8). **Table S1** showed the details of the three isocaloric diets (Dyets Inc., Bethlehem, PA, USA, Cat D211015, D211016, and D211017). After 2 weeks of feeding, the three groups (CCDI, PCDI, and ICDI) were modeled for *C. difficile* infection as previously described (Chen et al., 2008). The experimental scheme consisted of 5 days of antibiotic cocktail water including kanamycin (0.4 mg/ml), gentamicin (0.035 mg/ml), colistin (850 U/ml), metronidazole (0.215 mg/ml) and vancomycin (0.045 mg/ml). Clindamycin (10 mg/kg, ip, D -1) was administered after the next 2 days of normal water intake. The animals in the CNC group were injected intraperitoneally with phosphate-buffered saline (PBS), which was the vehicle control for clindamycin. On D0, all animals except those in the CNC group received 10^8^ CFU of *C. difficile*. The mice were observed, and disease symptoms and diarrhea were recorded. The mice were euthanized on D6.

### Histopathological analysis

The colon samples were embedded in paraffin and cut into 4 μm sections. Then, they were stained with hematoxylin and eosin (H&E), and histopathological scores were assessed according to a previous method (Chen et al., 2008). Goblet cells and the mucus layer were observed and evaluated by Alcian blue periodic acid Schiff (AB-PAS) staining. For immunofluorescence and immunohistochemical staining, the embedded colon sections were immunostained with antibodies against ZO-1, F4/80, and Ly6G according to the manufacturer’s protocol.

### Measurement of serum cytokine and endotoxin levels

Serum endotoxin lipopolysaccharide (LPS) levels were quantified using the limulus amebocyte lysate (LAL) (Hycult Biotech, USA) assay kit. The concentration of LPS-binding protein (LBP) was measured with an ELISA kit from Abcam (Cambridge, MA, United States). The levels of serum cytokines, including G-CSF, IL-1α, IL-6, TNF-α, IL-1β, and MIP-1α, were analyzed by a cytokine assay kit (Bio-Rad, CA, USA).

### qRT−PCR analysis

Colon tissue RNA was extracted using the RNeasy Plus Mini kit (Qiagen, CA, USA). RNA was reverse transcribed into cDNA using PrimeScript RT kits (Takara Biomedicals, Japan). mRNA expression was then repeatedly determined using the ViiA7 real-time PCR system (Applied Biosystems, Massachusetts, USA) with Premix Ex Taq (Takara Biomedicals). All gene expression levels were normalized to β-actin expression levels. Primer information is provided in **Table S2**.

### Sequencing of 16S rRNA

Fecal samples were collected prior to sacrificing the mice. The DNeasy Powersoil Pro Kit was used to extract DNA (Qiagen, Hilden, Germany). PCR amplification of the 16S rRNA gene was performed using modified primers. Sequencing was performed on the Illumina MiSeq platform (Gu et al., 2020). The raw tags were filtered according to QIIME, and chimeric sequences were removed by comparison with the Silva database. Operational taxonomic units (OTUs) were determined as sequences with at least 97% identity.

### Metabolic profiling

The cecal contents were collected and stored at -80°C. Metabolomic samples were prepared as described previously (Bian et al., 2020). Metabolites were analyzed by gas chromatography-mass spectrometry (GC−MS) using an Agilent 7890A GC system coupled with an Agilent 5975C inert mass selective detector system (Agilent Technologies, Santa Clara, CA). Metabolites were identified using Lumingbio’s untargeted GC−MS database. Partial least squares discrimination analysis (PLS-DA), orthogonal PLS-DA (OPLS-DA), and projection (VIP) values were used to calculate the significance of variables.

The SCFAs in the cecal contents were detected by GC−MS analysis of 20 mg of dry weight contents according to a previously described experimental procedure (Bian et al., 2020).

### AhR agonist and antagonist treatments

For the AhR agonist intervention, mice were administered 6-formylindolo [3,2-b] carbazole (FICZ; Sigma, Germany) by intraperitoneal injection (1 µg/mouse) once a week. For AhR antagonist intervention, mice were administered 2-methyl-2H-pyrazole-3-carboxylic acid (CH223191; Sigma, Germany) by intraperitoneal injection (10 µg/mouse) once a week (Monteleone et al., 2011).

### Statistical analysis

Data analysis was performed using GraphPad Prism v9.0.0. The data are presented as the mean ± the standard error of the mean (SEM). For the determination of statistical significance, a one-way analysis of variance (ANOVA) followed by Tukey’s test was used. *P* values < 0.05 indicated statistical significance.

## Results

### A pectin diet provides protection against *C. difficile* infection

To explore the effect of dietary fiber on CDI, we fed mice a diet (**Table S1**) containing 10% cellulose (CNC and CCDI groups), or three-quarters of the fiber was changed to inulin (INC and ICDI groups) or pectin (PNC and PCDI groups) (**Figure 1A**). After 2 weeks of feeding, CDI modeling was performed. All infected mice (CCDI, ICDI, and PCDI groups) showed typical symptoms of infection, while the uninfected mice in the control group (CNC, INC, and PNC) remained healthy, and no mice died or showed signs of infection during the experiment. Typical clinical symptoms of infection were significant weight loss (**Figure 1D**) and diarrhea. These effects were further exacerbated in the ICDI group, which showed weight loss and more diarrhea than in the CCDI group, but only mild symptoms were observed in the PCDI group. Survival rates differed significantly between the pectin-fed mice and the animals in all other infectious groups, and pectin protected the mice (**Figure 1C**). Proximal colon tissue showed a better appearance and colon length in the PCDI group than in the CCDI group (**Figure 1B**). Histopathological analysis showed significantly greater pathology in all infected mice than in uninfected mice (**Figure 1F**). Histological analysis showed that CCDI group and ICDI group mice exhibited typical features of colitis, including a thickened colon wall, distorted crypt structures, and infiltration of inflammatory cells in the mucosa. The pathology and scores in the PCDI group were significantly better than those in the CCDI and ICDI groups (**Figure 1E**). Goblet cells secrete mucus to protect the colon against pathogens. AB-PAS staining showed that the CCDI group exhibited significantly less mucus secretion and fewer goblet cells than the normal group (**Figure 1F**). The pectin dietary intervention significantly alleviated the *C. difficile*-induced reduction in mucus and goblet cells. Immunofluorescence staining of macrophages (F4/80+) and neutrophils (Ly6G+) in colon tissue was also performed, demonstrating that infection could cause the infiltration of macrophages and neutrophils in colon tissue and that pectin reduced this inflammatory phenomenon (**Figure 1F**).

### Pectin ameliorates intestinal barrier injury induced by *C. difficile* infection

**Figure 2:**
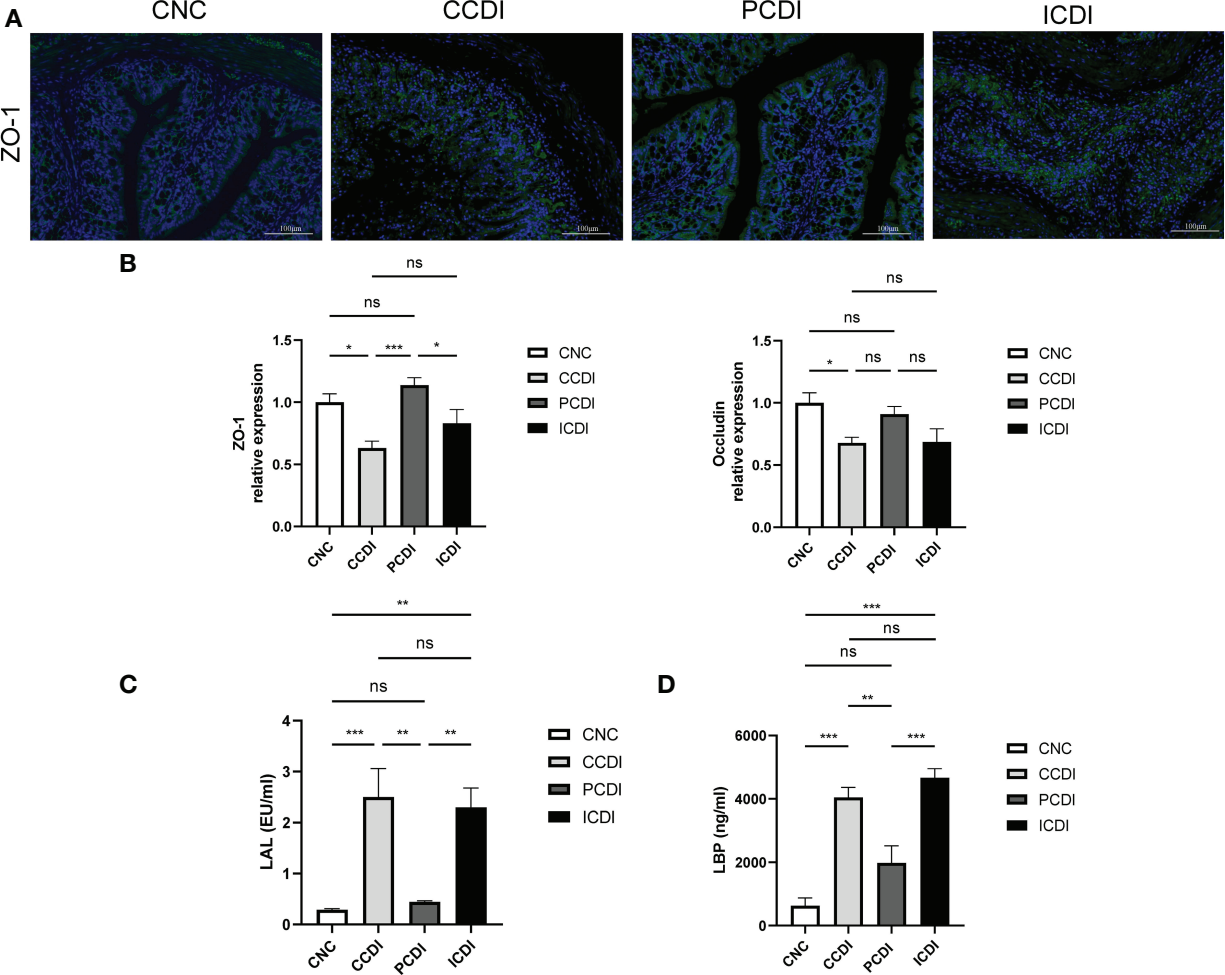
Pectin enhances intestinal barrier function. **(A)** Immunofluorescence staining of colon tissue. **(B)** Relative mRNA levels of ZO-1 and Occludin in colon tissues measured by qPCR. **(C, D)** Levels of the inflammatory markers LAL and LBP in mouse serum. *, *P*<0.05; **, *P*<0.01; ***, *P*<0.001; ns, differences are not significant. LAL, limulus amebocyte lysate; LBP, lipopolysaccharide-binding protein.

*C. difficile* produces toxins and following *C. difficile* infection, the intestinal barrier is defective. Intestinal barrier disruption leads to increased intestinal permeability and endotoxin translocation. To assess the bacterial translocation caused by increased intestinal permeability, we further examined the serum LAL and LBP levels (**Figures 2C, D**), and *C. difficile* infection led to increased serum LAL and LBP levels in the CCDI group, while the pectin diet decreased the endotoxin response of the organism. Thus, serum immunoreactivity to the bacterial product LPS was increased in both the CCDI and ICDI groups of mice. We also examined the results of immunofluorescence staining for the intestinal barrier protein ZO-1 and evaluated the expression of intestinal barrier indicators (ZO-1 and Occludin) by qPCR. We found that the intestinal barrier was compromised in the CCDI group, while pectin (PCDI group) protected *C. difficile*-infected mice against damage to the intestinal barrier (**Figure 2A**) and rescued the mRNA levels of tight junction proteins (Tjps) in colonic tissue (ZO-1 and Occludin, *P*<0.05; **Figure 2B**), while no alleviation was observed in the inulin group. These results indicate that pectin protects mice from infection *via* mucosal barrier enhancement.

### Pectin attenuates the serum and intestinal inflammatory response in *C. difficile* infection

**Figure 3:**
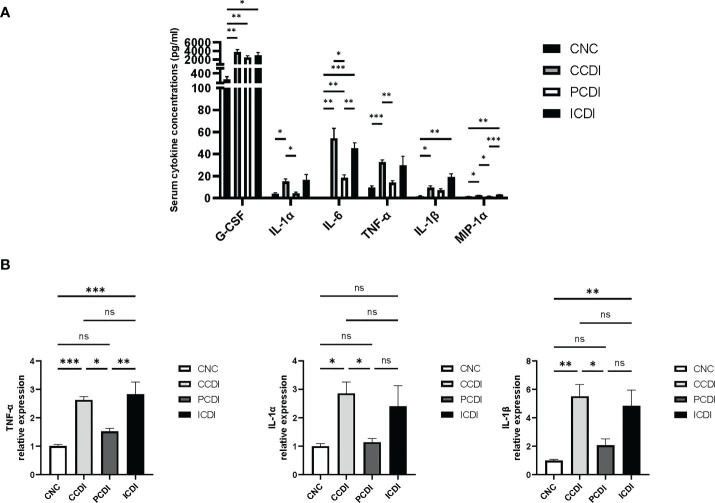
Pectin attenuates the systemic and intestinal inflammatory responses in mice with CDI. **(A)** Serum cytokine levels in the mice. **(B)** mRNA expression levels of TNF-α, IL-1α, and IL-1β in mouse colonic tissues. *, *P*<0.05; **, *P*<0.01; ***, *P*<0.001; ns, differences are not significant.

The production of toxins by *C. difficile* causes inflammation in the intestine (Kordus et al., 2022). We assessed the immune status by measuring the levels of cytokines reflecting immune cell signaling activity. The mRNA levels of serum cytokines and intestinal inflammatory factors were examined in *C. difficile*-infected mice fed different diets. The results showed that the levels of serum cytokines, such as G-CSF, IL-1α, IL-6, TNF-α, IL-1β, and MIP-1α, were higher in the CCDI group than in the CNC group (**Figure 3A**). Interestingly, the levels of inflammatory factors (IL-1α, IL-6, TNF-α, and MIP-1α) were decreased in the PCDI group (*P*<0.05). We also assessed the mRNA levels of inflammatory factors in intestinal tissues and showed that the expression of inflammatory biomarkers (TNF-α, IL-1α, and IL-1β) was higher in the CCDI group (**Figure 3B**) than in the CNC group, while the pectin diet improved the intestinal inflammation levels. In conclusion, these results suggest that colon inflammation induced by *C. difficile* infection was alleviated in the PCDI group but not significantly alleviated in the ICDI group.

### Different fibers have different effects on the gut microbiota

**Figure 4:**
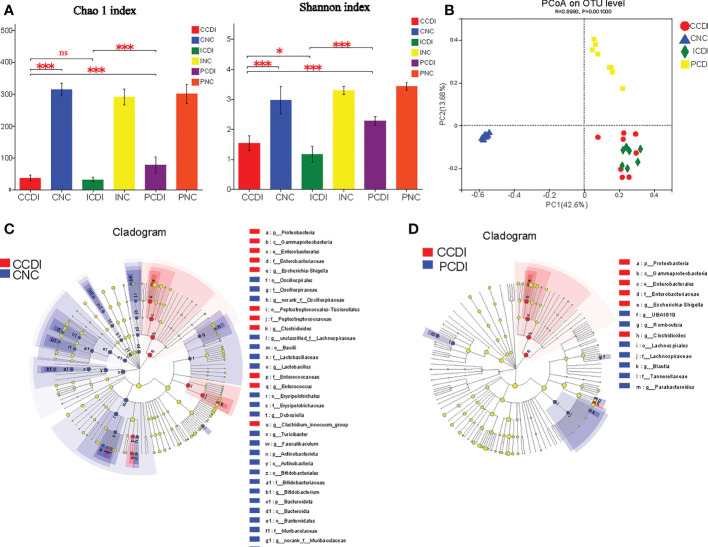
Pectin alleviates the dysbiosis of intestinal flora in CDI. **(A)** Chao1 and Shannon indices of intestinal flora. **(B)** The PCoA plot based on unweighted UniFrac shows the β-diversity of the gut microbiome. **(C, D)** LEfSe cladogram. Blue represents the CNC or PCDI group, and red represents the CCDI group. *, *P*<0.05; ***, *P*<0.001; ns, differences are not significant. LEfSe, linear discriminant analysis effect size.

Considering the different effects of inulin and pectin on the degree of colitis, we compared the microbiota composition that might underlie or correlate with such differences. To analyze the diet-induced changes in the intestinal flora, we used 16S rRNA sequencing to compare the stool samples (CNC=8, PNC=8, INC=8, PCDI=8, CCDI=8, and ICDI=7). Through comparison of the α-diversity (Chao1 and Shannon index), we found that *C. difficile* infection decreased gut microbial diversity, while pectin increased diversity (**Figure 4A**). β-diversity (PCoA based on unweighted UniFrac) indicated differences in gut flora structure between groups (**Figure 4B**).

To identify the characteristic microorganisms, we performed linear discriminant analysis effect size (LEfSe) analysis. LEfSe showed that the CCDI group exhibited higher abundances of opportunistic pathogenic bacteria, such as *Enterobacteriaceae*, *Peptostreptococcaceae*, and *Enterococcaceae*, and lower abundances of *Lactobacillaceae*, *Bifidobacteriaceae*, *Muribaculaceae*, *Rikenellaceae*, *Akkermansiaceae*, and *Desulfovibrionaceae* at the family level than the CNC group (**Figure 4C** and **Figure S1**). The relative abundance of *Enterobacteriaceae* was lower in the PCDI group than in the CCDI group (**Figure 4D**). We observed higher abundances of *Lachnospiraceae* and *Blautia* in the PCDI group than in the CCDI and the ICDI groups; these bacteria reduce intestinal inflammation and promote intestinal health (**Figure 4D** and **Figure S2**). Bacteria known to readily metabolize fibers into SCFAs, including *Lachnospiraceae*, are preferentially enhanced by pectin (Riviere et al., 2016). Our results show that the pectin diet increased the abundance of these SCFA-producing bacteria over the inulin diet.

### Distinct fibers differentially impact the composition of metabolites

**Figure 5:**
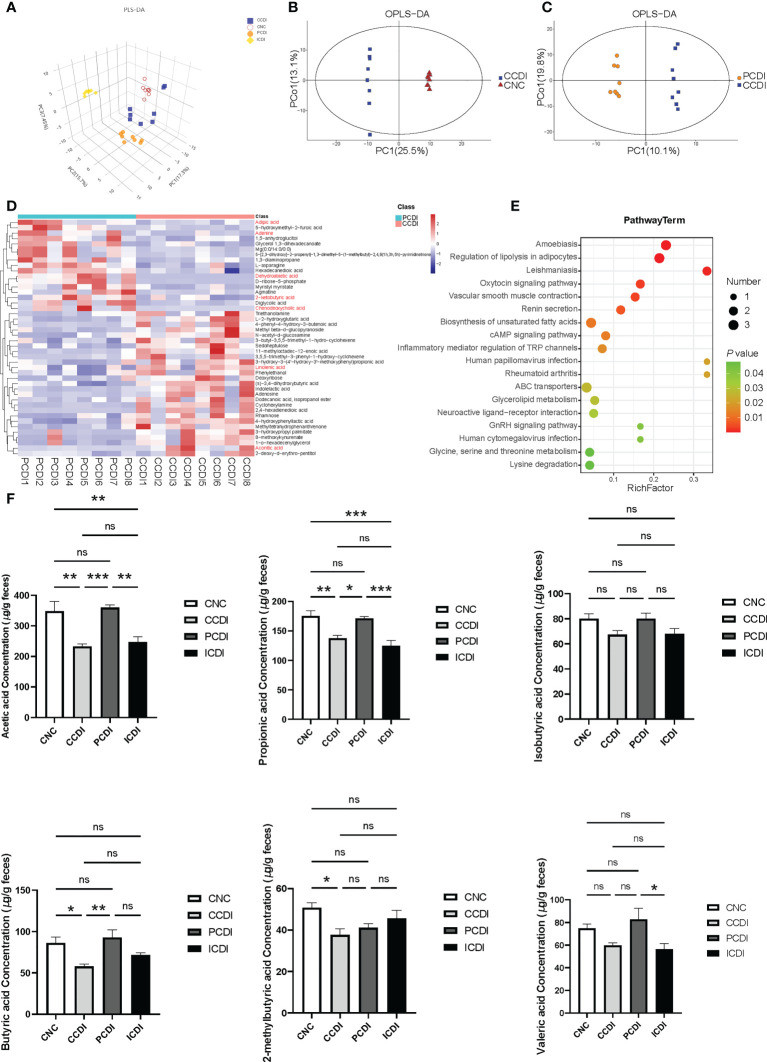
Pectin attenuates *C*. *difficile*-induced dysregulation of the intestinal metabolome. **(A)** Three-dimensional PLS-DA score plots of metabolome profiles for the four groups. **(B, C)** OPLS-DA plots between groups. **(D)** Heatmap for the selected metabolites in the CCDI and PCDI groups. **(E)** Pathway of the differentially abundant metabolites between the CCDI and PCDI groups. **(F)** SCFA levels in the cecal contents of mice. *, *P*<0.05; **, *P*<0.01; ***, *P*<0.001; ns, differences are not significant. PLS-DA, Partial least squares discrimination analysis; OPLS-DA, orthogonal PLS-DA. The red color showed valuable differential metabolites.

To explore the relationship between changes in intestinal metabolites and *C. difficile* intestinal damage, we performed metabolomic analysis using GC−MS analysis of the cecal contents obtained from the four groups. A total of 632 metabolites were identified. The PLS-DA plot showed that each group’s metabolomic profile was distinct (**Figure 5A**), indicating that the metabolomic composition of these groups was different. OPLS-DA plots showed the differences in metabolomic composition between the CCDI group and the CNC group and between the PCDI group and the CCDI group (**Figures 5B, C**).

Using VIP>1 (based on the OPLS-DA model) and *P*<0.05 between groups, we further explored the different metabolic profiles in the PCDI and CCDI groups. The heatmap showed that the characteristic metabolites between the PCDI and CCDI groups were mainly related to carbohydrates, amino acids, lipids, etc. (**Figure 5E**). The differential metabolic pathways included the biosynthesis of unsaturated fatty acids, the cAMP signaling pathway, glycine, serine and threonine metabolism, and inflammatory mediator regulation of TRP channels. The characteristic metabolites in the two groups are shown (**Figure 5D**). The PCDI group was associated with relatively higher levels of the amino acids and bile acids required to inhibit the germination and growth of *C. difficile*, such as chenodeoxycholic acid, than the CCDI group. The PCDI group also showed reduced levels of linolenic acid and aconitic acid. Dehydroabietic acid, adenine, adipic acid, and 2-ketobutyric acid levels were relatively increased in the PCDI group (**Figure 5D**). We also compared and analyzed the different metabolic profiles that may lead to different efficacies of pectin and inulin diets. Compared to the ICDI group, the PCDI group reduced carbohydrates (e.g. trisaccharide, maltotriose, erythrose, gluconic acid, and galactitol) along with amino acids (valyl-glycine, alanyl-threonine, L-proline, and valyl-valine). In contrast, the PCDI group increased L-aspartic acid, tartaric acid, spermidine, L-phenylalanine, and cholic acid (**Figure S3**).

Pectin is known to affect the SCFAs involved in T-cell immunity (Smith et al., 2013). To explore whether the protective effect of dietary fibers against infection is related to the major products of fiber metabolism of the microbiome, such as SCFAs, we examined the SCFAs, such as acetic acid, propionic acid, isobutyric acid, butyric acid, 2-methylbutyric acid, and valeric acid, in mouse cecal contents. In the CCDI group, *C. difficile* infection reduced the levels of acetic acid, propionic acid, butyric acid, and 2-methylbutyric acid (*P*<0.05, **Figure 5F**). The concentrations of acetic acid, propionic acid, and butyric acid were higher in the PCDI group than in the CCDI group (*P*<0.05, **Figure 5F**). The levels of acetic acid were higher in animals fed the pectin diet than in those fed the inulin diet (**Figure 5F**). Furthermore, we conducted a correlation analysis between SCFAs and *Lachnospiraceae* (**Figure S4**). The results exhibit a good linear correlation between the relative abundance of *Lachnospiraceae* and SCFAs (such as acetic acid and butyric acid).

### Pectin protects against *C. difficile* infection by activating the AhR pathway

**Figure 6:**
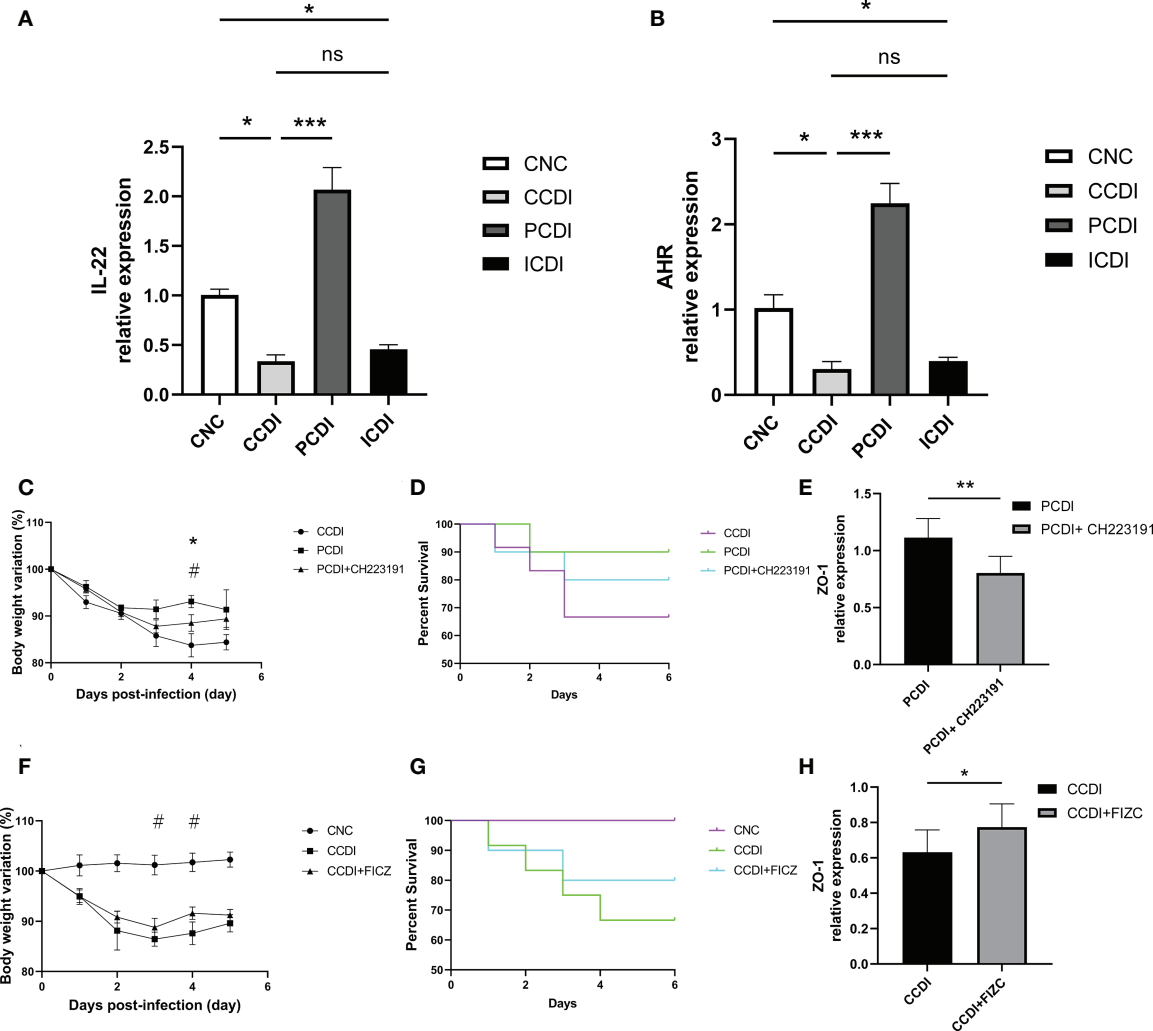
Pectin protects mice with CDI from intestinal infection by enhancing AhR pathway activation. **(A, B)** Relative mRNA levels of IL-22 and AhR in colon tissue. *, *P*<0.05; **, *P*<0.01; ***, *P*<0.001; ns, differences are not significant. **(C, D)** Body weight changes and survival curves of the mice with CDI treated with the AhR antagonist CH223191. *, CCDI vs PCDI+ CH223191, *P*<0.05; ^#^, PCDI vs PCDI+ CH223191, *P*<0.05. **(E)** Relative mRNA levels of ZO-1 in colon tissues. **(F, G)** Body weight changes and survival curves of the mice with CDI treated with the AhR agonist FICZ. ^#^, CCDI vs CCDI+FIZC, *P*<0.05. **(H)** Relative mRNA levels of ZO-1 in colon tissues.

Previous studies have shown that catabolic substances produced by the microbiota can modulate interleukin (IL)-22 production and boost T-cell immunity by activating AhR (Ye et al., 2017; Lamas et al., 2018), which plays a role in mucosal immunity (Zelante et al., 2013). Previous studies have shown that pectin increases the production of metabolites by the microbiota to activate AhR (Monteleone et al., 2011), thereby improving intestinal barrier function. Our gut microbiota analysis also showed that pectin treatment increased *Lachnospiraceae* abundance in the intestine, which is also involved in the activation of the AhR pathway by metabolic substances (Vacca et al., 2020; Zhang et al., 2021). We next determined the role of the AhR pathway in the pectin treatment of *C. difficile*-induced severe colitis. IL-22, secreted by CD3+ T cells and ILC3s in the intestine, has been shown to protect the host from infection as a downstream gene of the AhR pathway (Parks et al., 2015). We evaluated the expression levels of the relevant indicators (AHR and IL-22) in colon tissues of different groups by qPCR (**Figures 6A, B**). The results showed that the AhR and IL-22 mRNA levels were lower in the CCDI group than in the CNC group, while the pectin group showed increased levels.

We also explored the targeted therapeutic strategies of the AhR pathway in CDI using AhR agonists and inhibitors. The body weight in the *C. difficile*+pectin+AhR antagonist group was lower than that in the *C. difficile*+pectin group (**Figure 6C**), indicating that the AhR antagonist exacerbated the infection and clinical symptoms in the pectin diet-fed mice. The death of the pectin-treated mice was accelerated early after *C. difficile* induction because of the AhR antagonist (**Figure 6D**), but the effect of pectin pretreatment on the survival of the *C. difficile*-infected mice could not fully counteract the outcome. Next, the effect of the AhR agonist on *C. difficile* infection was evaluated. Body weight loss was lower in the *C. difficile*+AhR agonist-treated mice than in the *C. difficile*-treated mice (**Figure 6F**). AhR agonists increased the survival rate of infected mice (**Figure 6G**). We also assessed the expression of intestinal barrier indicators such as ZO-1, and the results showed that the AhR antagonist diminished the protective effect of pectin (**Figures 6E, H**). In conclusion, these findings suggest that the roles of gut microbiota in infection after pectin treatment are partly mediated by the AhR pathway. These results confirm that the AhR-dependent pathway contributes to pectin diet-mediated protection against *C. difficile* infection in mice.

## Discussion

The microbiota in the gut plays a crucial role in host health and provides resistance to a variety of intestinal pathogens (Sassone-Corsi and Raffatellu, 2015; Khan et al., 2022). The depletion of the gut microbiota caused by antibiotics can be exploited by pathogens such as *C. difficile* (Kriss et al., 2018). Diet affects the composition and function of the gut microbiota. Adequate intake of dietary fiber may play a beneficial role in enhancing intestinal immunity by regulating the gut microbiota (Makki et al., 2018). The study showed the effects of dietary composition on the physiology and pathogenesis of *C. difficile* in an animal model of antibiotic-induced CDI. Our study extensively assessed the functions of two soluble fibers, pectin and inulin, on a mouse model and the response of microbial communities to a diet with a widely varying nutrient composition following antibiotic treatment. Pectin was shown to improve the clinical outcomes of infection compared to inulin, and the mechanism underlying the relief in *C. difficile* infection may relate to anti-inflammatory effects, protection of the mucosal barrier, and maintenance of intestinal flora and metabolism homeostasis. Our study showed that inulin did not exhibit effective protection. However, a previous study showed a protective effect of inulin against *C. difficile* infection (Hryckowian et al., 2018). The different roles played by carbohydrates in *C. difficile* infection may be related to the type of carbohydrate and its concentration. The ability to target the microbiome through diet would enable microbiome-based modulation to ameliorate *C. difficile* infection.

Carbohydrate-rich diets (especially high-fiber diets) improve gut health, which has been well studied and is thought to be associated with SCFA production by intestinal flora (Maslowski et al., 2009; Singh et al., 2014). Several studies have shown that dietary fiber alleviates *C. difficile* infection (Hryckowian et al., 2018). *C. difficile* is directly affected by diet through germination, growth, and spore formation as well as indirectly by ecological interactions. In addition, diet may directly alter pathogenic factors expressed by *C. difficile*. Several studies have shown that pectin beneficially affects immunity and prevents inflammation and disease, and it can improve colon cancer by modulating signaling pathways activated by oxidative stress and inflammation (Tan et al., 2018). Toxins produced by *C. difficile* are thought to be the main virulence factors. These toxins induce inflammation, intestinal damage, and diarrhea (Kordus et al., 2022). Our experimental results showed that a pectin diet protected the intestinal barrier, reduced the cellular permeability of intestinal epithelial cells, and reduced the elevated LBP and LPS levels due to the impaired intestinal barrier caused by *C. difficile* infection. The etiology and pathogenesis of *C. difficile* infection are complicated, and an imbalance of pro- and anti-inflammatory cytokines has been linked to *C. difficile* development and progression, leading to persistent inflammatory response in the colon (Pawlowski et al., 2010). Our experimental results showed significantly higher cytokine levels in the CCDI group, and the pectin diet improved this outcome.

The gut microbiome contributes to the disease susceptibility and outcome of CDI and impacts innate and adaptive immunity (Li and Chen, 2022). Consistent with previous studies (Schubert et al., 2015; Ross et al., 2016; Wu et al., 2022), *C. difficile* infection is associated with a flora imbalance and decreased diversity of intestinal microbes. Our results showed that *C. difficile* reduced microbiome richness and diversity, whereas pectin improved microbiome flora diversity. There is clearly a link between microbiome status and disease regulation. Microorganisms can take bile acids from the liver into the intestine and convert them into secondary bile acids. The primary to secondary bile acid ratio is critical and affects the growth of *C. difficile*. *C. difficile* also produces toxins that penetrate the intestinal barrier, while SCFAs, produced by other intestinal microbes, help tighten the intestinal barrier. The maintenance of the homeostasis of the intestinal flora is closely related to the number of bacteria that produce SCFAs, which contribute to the maintenance of the homeostasis of the intestinal environment. Other methods to decrease *C. difficile* survival involve nutritional competition for ecological niches in the intestinal environment and other indirect mechanisms. *C. difficile* infection leads to an increase in the abundance of intestinal opportunistic pathogens such as *Escherichia-Shigella* and *Enterococcus* (Gu et al., 2016). Opportunistic pathogens promote intestinal inflammation and are triggered to induce disease by intestinal inflammation. In contrast, a pectin diet increases the level of beneficial intestinal flora, such as *Lachnospiraceae*, which are the main producers of butyrate in the intestine (Baxter et al., 2019). Correlation analysis showed a good correlation between *Lachnospiraceae* and SCFAs in our results. The pectin group, but not the inulin group, increased the abundance of *Lachnos*piraceae, which may contribute to the observed differences in treatment outcomes between the two diets. *Lachnospiraceae* also produce indole derivatives, which are tryptophan-converting metabolites that activate AhR (Vacca et al., 2020). Rorγt^+^ Tregs, which are highly expressed in the intestinal tract, may be stimulated by these tryptophan metabolites (Ye et al., 2017). We also used AhR agonists and inhibitors to demonstrate the involvement of the AhR pathway in the role of a pectin diet against *C. difficile* infection.

Numerous intestinal commensal metabolites, including amino acid derivatives, carbohydrates, and vitamins, modulate a variety of host immune cell subsets through different mechanisms (Li et al., 2022). Based on these findings, we hypothesized that a fermentable fiber diet would negatively affect *C. difficile* adaptation in two interrelated ways. First, a fermentable fiber diet promotes the privileged growth of members of the microbiota that utilize fiber (e.g., *Bacteroides*). Second, SCFAs produced by fermentable fiber metabolism negatively impact *C. difficile* adaptation, likely due to the accumulation of key metabolic pathway end products, such as reduced acetate production and butyrate production (Ferreyra et al., 2014). Dietary fiber and its products, particularly SCFAs, have been proven to benefit inflammatory diseases (Maslowski et al., 2009; Trompette et al., 2014). The expression of TcdB and TcdA is modulated by many factors, such as nutrients, population sensing, and other environmental indices. SCFAs act as signals from the microbiome to ferment *C. difficile*, and this competitive and unsuitable intestinal environment can lead to increased toxin production. *C. difficile* strains are inhibited by SCFAs based on concentration (Hryckowian et al., 2017). Butyrate can affect toxin expression (Karlsson et al., 2000). Butyrate also protects against CDI by protecting the intestinal barrier and alleviating inflammation through overexpression of hypoxia-inducible factor 1 (HIF-1), the host pathways that may independently affect inflammation and *C. difficile* burden and toxin (Kelly et al., 2015; Fachi et al., 2019). Moreover, gut microbes play a role in bile acid metabolism, affect *C. difficile* (Buffie et al., 2015) and may interact with dietary influences. These metabolic processes convert primary bile acids and conjugated bile acids (e.g., taurocholic acid), which promote *C. difficile* germination, into unconjugated primary bile acids and secondary bile acids (e.g., cholic acid and deoxycholic acid), which are either less effective germination agents or even inhibit this process (Ridlon et al., 2006). Our results showed that the pectin group modulated bile acids such as chenodeoxycholic acid, which may also be one of the mechanisms underlying the effects of pectin on CDI. Bile acids are an important signal for *C. difficile* spore germination; however, the bile acid signal alone is not sufficient. Amino acids such as glycine are another signal necessary for *C. difficile* spore germination. Glycine is an important germinator of *C. difficile* spores (Neumann-Schaal et al., 2019). Glycine, serine, and threonine metabolism is one of the differential metabolic pathways after pectin treatment. By comparing the metabolomes of the pectin and inulin groups, the different metabolites, such as l-phenylalanine, had an inhibitory effect on *C. difficile* spore growth (Pickering et al., 2018). And valine is also a co-emergent source of *C. difficile*. More work will focus on exploring how dietary factors may influence the dynamics of infection.

The gut microbiota has been identified as a potential modifiable nongenetic factor. Diet can affect the microbiota, supporting the hypothesis that changes in diet may affect the occurrence and development of *C. difficile* infection. Recently, studies evaluated the consumption of fiber foods rather than refined fiber, and we examined the effect of refined fibers (pectin and inulin) on *C. difficile* infection. We observed that dietary pectin prevented the development of *C. difficile*-induced colitis, whereas inulin promoted its development, probably at least in part by promoting the activation of the AhR pathway. Although fiber has many beneficial effects on the gut, it may also have negative effects, which may depend on the genetic and physiological status of the host. Accordingly, pectin can be conditionally beneficial, depending on preexisting intestinal conditions and the specific fiber. According to these results, specific dietary fibers may have benefits or risks in the case of *C. difficile* infection. Limitations of this experiment include the fact that our study focused on the effects of pectin and inulin on *C. difficile* infection, used cellulose as a control group, and did not set an infectious group with a standard lab diet, which will be verified in our subsequent experiments.

Our results support the idea that certain dietary fibers have the potential to attenuate inflammation, and the utilization of dietary fiber flora and associated end products of dietary fiber metabolism, such as SCFAs, are associated with reduced *C. difficile* adaptations. Current microbiota-centered therapies for *C. difficile* infection, such as fecal microbiota transplantation and probiotics, are mediated by the introduction of exogenous microorganisms. Prebiotics, such as pectin, is a promising approach to improve the human microbiota. Our studies suggest that different fibers can have different effects, which may also be due to the different microbial fermentations of different fibers. A better understanding of the fibers may contribute to the optimization of fiber-based personalized treatments for *C. difficile* infection.

## Data availability statement

The datasets presented in this study can be found in online repositories. The names of the repository/repositories and accession number(s) can be found below: https://www.ncbi.nlm.nih.gov/, PRJNA 873028.

## Ethics statement

The animal study was reviewed and approved by the Animal Experimental Ethical Inspection of The First Affiliated Hospital, Zhejiang University School of Medicine.

## Author contributions

LJL and QX designed the experiments. QW and QX analyzed the data. ZW wrote the manuscript. Revision of the manuscript by other authors. All authors contributed to the article and approved the submitted version.

## Funding

This work was supported by the National Natural Science Foundation of China (82000491, 82073609, 81790631); the National Key Research and Development Program of China (2018YFC2000500); Research Project of Jinan Microecological Biomedicine Shandong Laboratory (JNL-2022001A); the Fundamental Research Funds for the Central Universities (2022ZFJH003).

## Conflict of interest

The authors declare that the research was conducted in the absence of any commercial or financial relationships that could be construed as a potential conflict of interest.

## Publisher’s note

All claims expressed in this article are solely those of the authors and do not necessarily represent those of their affiliated organizations, or those of the publisher, the editors and the reviewers. Any product that may be evaluated in this article, or claim that may be made by its manufacturer, is not guaranteed or endorsed by the publisher.
