# Supplementary material for: The impact of dietary fibers on Clostridioides difficile infection in a mouse model
Source: Front Cell Infect Microbiol. 2022 Nov 9;12:1028267. doi: 10.3389/fcimb.2022.1028267 (PMC9682084; doi:10.3389/fcimb.2022.1028267)
Supplement: Supplementary file 1 [file DataSheet_1.docx]

Supplementary Material

# Supplementary Figures and Tables

For more information on Supplementary Material and for details on the different file types accepted, please see [here](http://home.frontiersin.org/about/author-guidelines#SupplementaryMaterial).

## Supplementary Tables

**Supplementary Table S1. Diet composition (Dyets Inc.)**

| **Diet formulas** | **Cellulose containing diet (CCD)** | **Pectin containing diet (PCD)** | **Inulin containing diet (ICD)** |
| --- | --- | --- | --- |
| **Product #** | **D211016** | **D211017** | **D211015** |
|  | **gm** | **gm** | **gm** |
| **Caisein** | **200** | **200** | **200** |
| **L-Cystine** | **3** | **3** | **3** |
| **Sucrose** | **150** | **150** | **150** |
| **Dyetrose** | **110** | **110** | **110** |
| **Cornstarch** | **409** | **381** | **381** |
| **Soybean Oil** | **70** | **70** | **70** |
| **Cellulose** | **100** | **25** | **25** |
| **Pectin** | **0** | **75** | **0** |
| **Inulin** | **0** | **0** | **75** |
| **Mineral Mix #210088** | **10** | **10** | **10** |
| **Calcium Carbonate** | **5.5** | **5.5** | **5.5** |
| **Dicalcium Phosphate** | **13** | **13** | **13** |
| **Potassium Citrate H2O** | **16.5** | **16.5** | **16.5** |
| **Vitamin Mix #300050** | **10** | **10** | **10** |
| **Choline Bitartrate** | **2** | **2** | **2** |
| **Blue Dye** | **0.05** | **0.025** | **0** |
| **Red Dye** | **0** | **0.025** | **0.05** |
| **TOTAL** | **1099.05** | **1071.05** | **1071.5** |

**Supplementary Table S2. PCR primers used for the RT-PCR analyses.**

| **Gene** | **Forward Sequence (5'-3')** | **Reverse Sequence (5'-3')** |
| --- | --- | --- |
| **β-actin** | **AGTGTGACGTTGACATCCGT** | **GCAGCTCAGTAACAGTCCGC** |
| **Occludin** | **TTCCTCTGACCTTGAGTGTGG** | **CTCTTGCCCTTTCCTGCTTT** |
| **ZO-1** | **GCCGCTAAGAGCACAGCAA** | **GCCCTCCTTTTAACACATCAGA** |
| **TNF-α** | **CAGGCGGTGCCTATGTCTC** | **CGATCACCCCGAAGTTCAGTAG** |
| **IL-1α** | **CGAAGACTACAGTTCTGCCATT** | **AAACTTCTGCCTGACGAGCTT** |
| **IL-1β** | **TGGACCTTCCAGGATGAGGACA** | **GTTCATCTCGGAGCCTGTAGTG** |

**2.1 Supplementary Figures**

**Supplementary Figure S1.** The dysbiosis of intestinal flora in CDI. (A) the bacterial taxa of the CCDI group were compared with those of the CNC group at different levels (LDA score > 4).

**
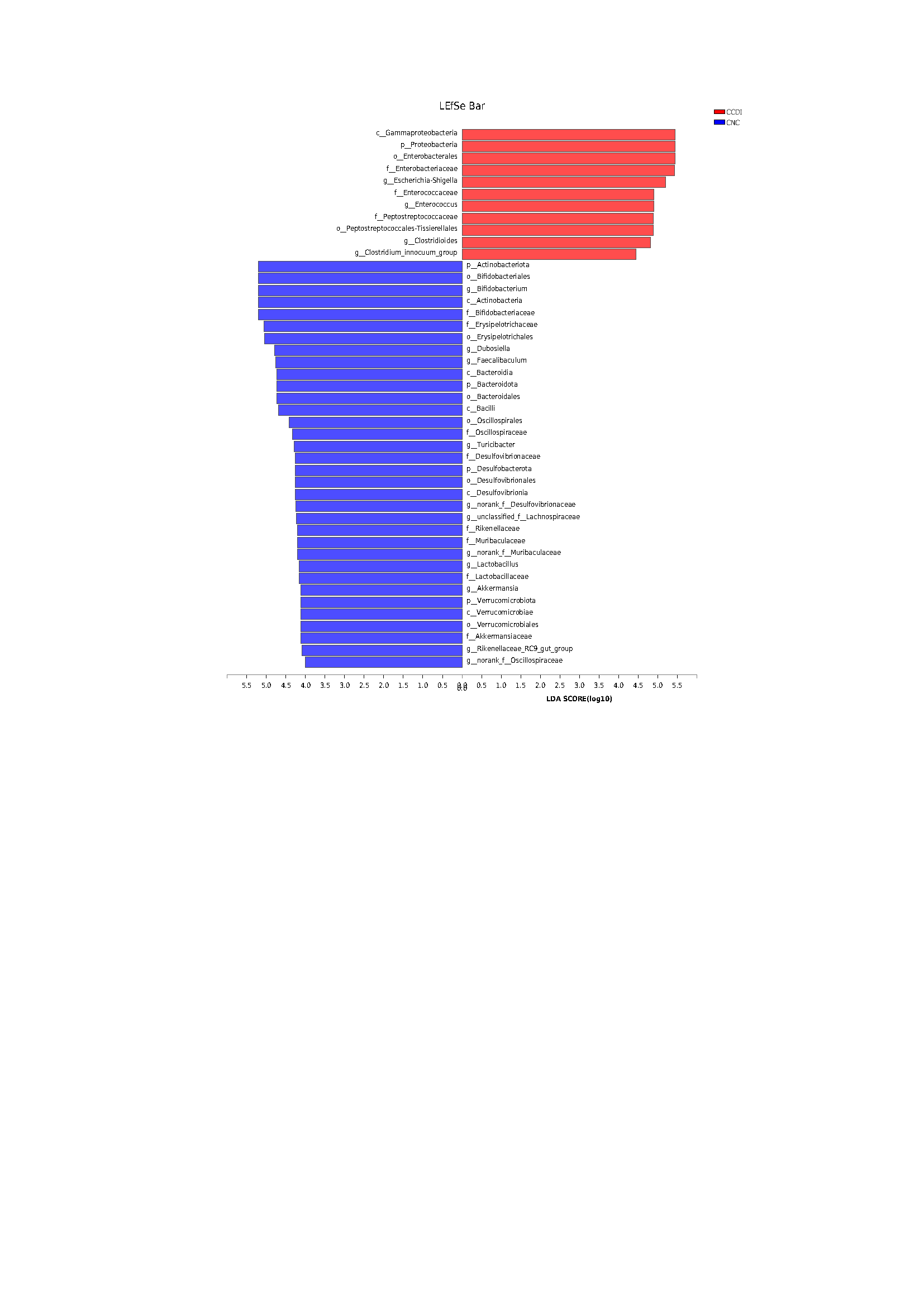
**

**Supplementary Figure S2.** Pectin alleviates the dysbiosis of intestinal flora in CDI. (A) the bacterial taxa of the CCDI group were compared with those of the PCDI group at different levels (LDA score > 4). (B) the bacterial taxa of the PCDI group were compared with those of the ICDI group at different levels (LDA score > 4).
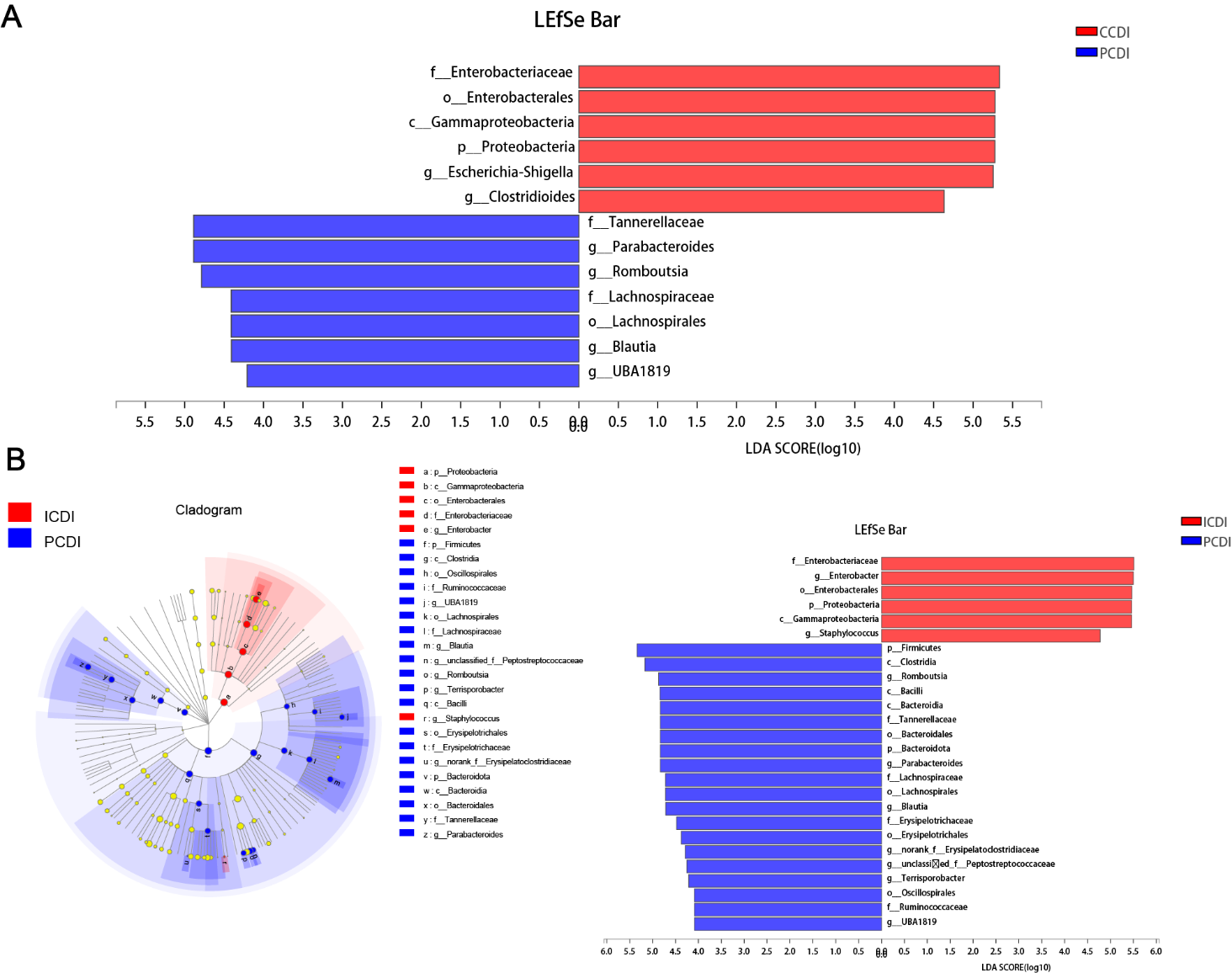


**Supplementary Figure S3.** Heatmap for the selected metabolites in the ICDI and PCDI groups. The red color showed valuable differential metabolites.

**
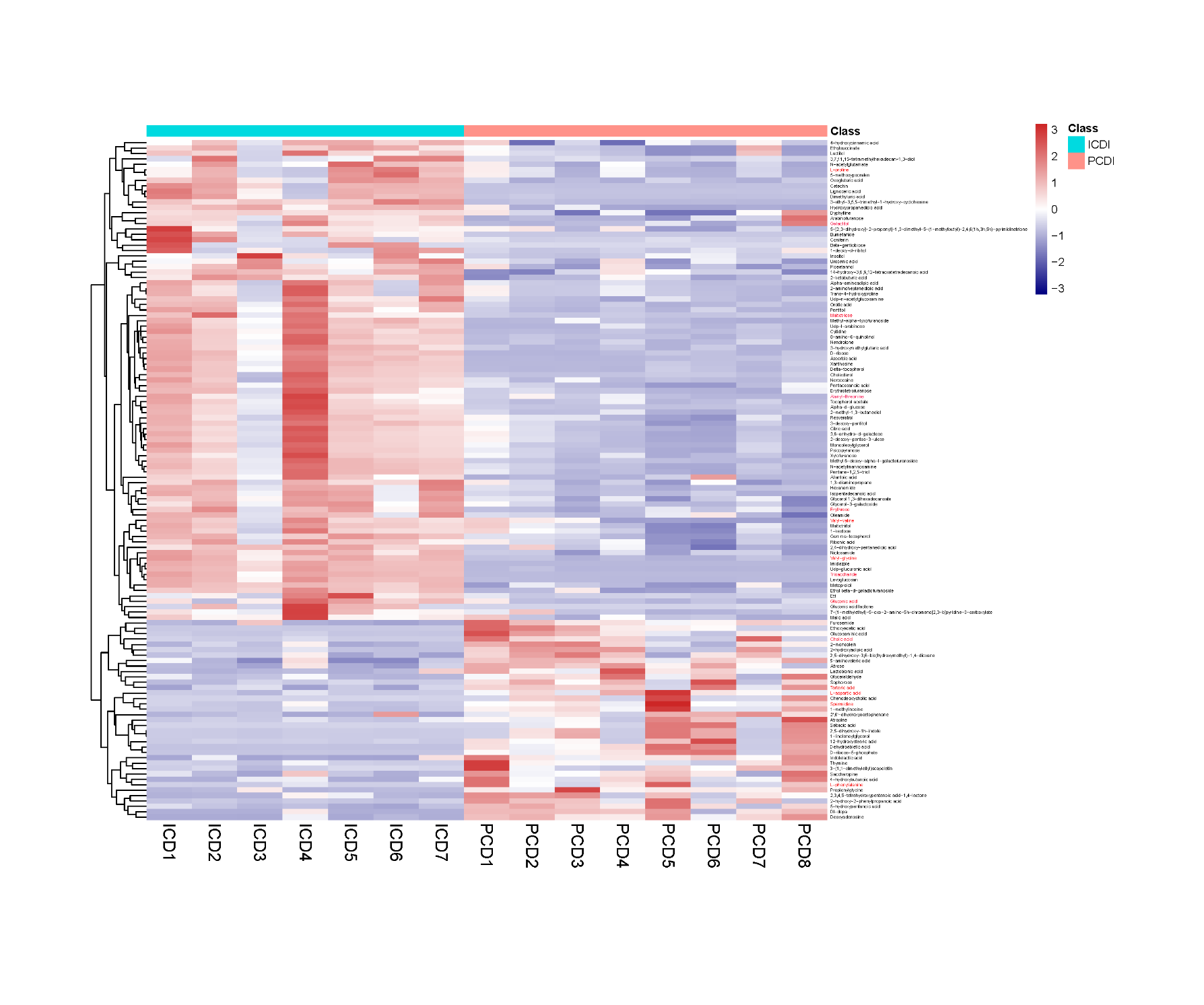
**

**Supplementary Figure S4.** Graph representative of correlation analysis. (A) acetic acid and the relative abundances of *Lachnospiraceae*. (B) butyric acid and the relative abundances of *Lachnospiraceae*.
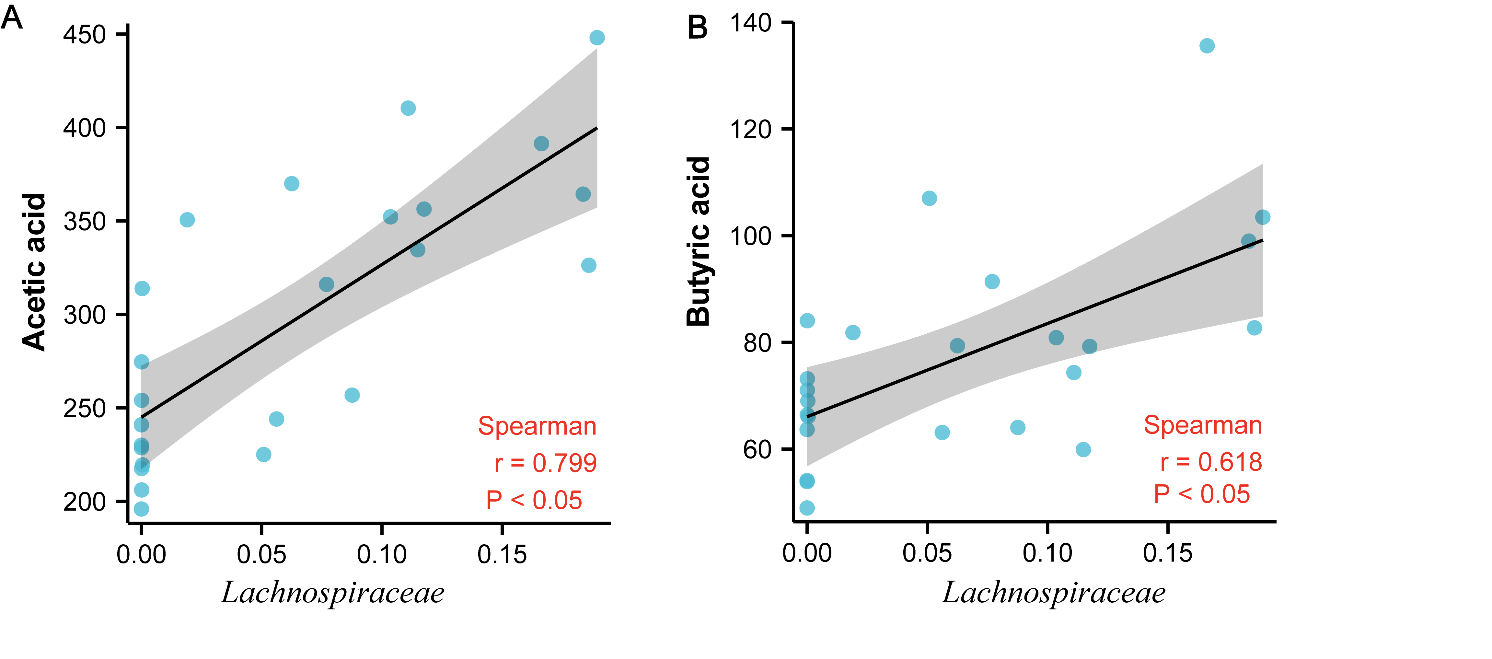


**
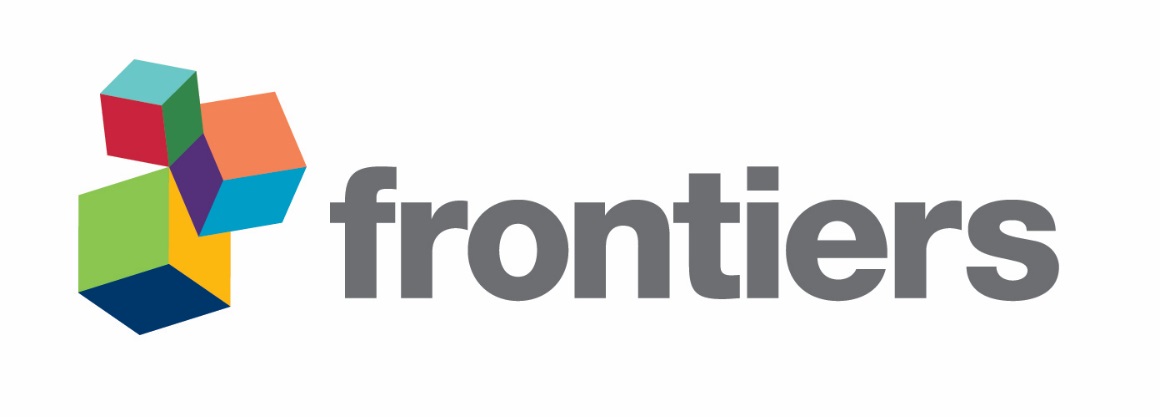
**
